# Efficient Mining of Anticancer Peptides from Gut Metagenome

**DOI:** 10.1002/advs.202300107

**Published:** 2023-06-29

**Authors:** Yue Ma, Xiaolin Liu, Xuan Zhang, Ying Yu, Yujing Li, Moshi Song, Jun Wang

**Affiliations:** ^1^ CAS Key Laboratory of Pathogenic Microbiology and Immunology Institute of Microbiology, Chinese Academy of Sciences 100101 Beijing P. R. China; ^2^ University of Chinese Academy of Sciences Beijing 100049 P. R. China; ^3^ Max Planck Institute for Evolutionary Biology 24306 Plön Germany; ^4^ State Key Laboratory of Membrane Biology Institute of Zoology Chinese Academy of Sciences 100101 Beijing P. R. China; ^5^ Institute for Stem Cell and Regeneration Chinese Academy of Sciences 100101 Beijing P. R. China; ^6^ Beijing Institute for Stem Cell and Regenerative Medicine 100101 Beijing P. R. China

**Keywords:** anticancer peptides, cancer therapy, gut microbiome, multi‐center mining, tumour inhibitors

## Abstract

The gut microbiome plays a crucial role in modulating host health and disease. It serves as a vast reservoir of functional molecules that hold great potential for clinical applications. One specific area of interest is identifying anticancer peptides (ACPs) for innovative cancer therapies. However, ACPs discovery is hindered by a heavy reliance on experimental methodologies. To overcome this limitation, we here employed a novel approach by leveraging the overlap between ACPs and antimicrobial peptides (AMPs). By combining well‐established AMP prediction methods with mining techniques in metagenomic cohorts, a total of 40 potential ACPs is identified. Out of the identified ACPs, 39 demonstrated inhibitory effects against at least one cancer cell line, exhibiting significant differences from known ACPs. Moreover, the therapeutic potential of the two most promising peptides in a mouse xenograft cancer model is evaluated. Encouragingly, the peptides exhibit effective tumor inhibition without any detectable toxic effects. Interestingly, both peptides display uncommon secondary structures, highlighting its distinctive characteristics. This findings highlight the efficacy of the multi‐center mining approach, which effectively uncovers novel ACPs from the gut microbiome. This approach has significant implications for expanding treatment options not only for CRC, but also for other cancer types.

## Graphical abstract

A workflow of discovering anticancer peptides (ACPs) from gut microbiome is devised, combining deeplearning based functional peptides identification and differential analysis in metagenomic data of colorectal cancer patients versus healthy. A total of 39 ACPs is found. The two most potent candidates are safe toward normal cells, with no acute toxicity or significantly reduced tumor growthin animals.

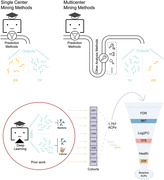

## 1 Introduction

The importance of the gut microbiome in maintaining host health and its involvement in the development of diseases are increasingly recognized. Dysbiosis of the gut microbiota has been implicated in metabolic disorders, autoimmune diseases,^[^ ^1^ ^]^ and various gastrointestinal cancers, particularly colorectal cancer (CRC).^[^ ^2^ ^]^ Moreover, emerging evidence suggests that that dysbiosis of the gut microbiota also contributes to non‐gastrointestinal tumors, including breast cancer,^[^ ^3^ ^]^ prostate cancer,^[^ ^4^ ^]^ and others. While previous studies have predominantly focused on pro‐inflammatory gut species like *Escherichia coli* and oncogenic molecules, such as colibactin,^[^ ^5^ ^]^ it is noteworthy that the gut microbiome can also impact cancer therapies by metabolizing medications^[^ ^6^ ^]^ or influencing the host's immune response.^[^ ^7^ ^]^ Consequently, there is growing interest in exploring interventions to modulate the gut microbiome as potential cancer treatments,^[^ ^8^, ^9^ ^]^ yielding promising outcomes. Notably, fecal microbiota transplantation has been demonstrated to enhance the response rate to anti‐PD‐1 immune checkpoint therapies.^[^ ^10^ ^]^ Furthermore, in animal models, gut fecal transplantation from ring finger protein 5 (Rnf5) knock out mice to germ‐free mice has effectively inhibited tumor development.^[^ ^11^ ^]^

The influence of the gut microbiome on host health and cancer development is mediated through a diverse array of macro‐molecules and small molecule metabolites.^[^ ^12^ ^]^ Within the gut microbiome, the extraordinarily high phylogenetic diversity leads to the production of a vast and varied pool of macro‐molecules, including polysaccharides, lipopolysaccharides, and peptidoglycans, resulting from complex enzymatic reactions. Additionally, the gut microbiome encodes peptides, proteins, and regulatory RNAs within its genomic sequences. Notably, a growing number of these molecules have shown therapeutic potential. For example, indole‐3‐lactic acid, produced by gut *Lactobacillus gallinarum*, has been found to suppress CRC growth both in vitro and in vivo.^[^ ^13^ ^]^ Similarly, reuterin, a metabolite generated by *Lactobacillus reuteri*, has recently been demonstrated to inhibit cancer development by disrupting reactive oxygen species in cancer cells.^[^ ^14^ ^]^ However, a significant proportion of small functional proteins and peptides with therapeutic potential remain inadequately characterized in relation to their role in human diseases. The diverse lengths and low sequence similarity of these molecules have presented challenges in their discovery, although recent advancements in deep learning approaches have facilitated progress in this area.

The application of deep learning in high‐throughput analysis of metagenomic data has proven to be an efficient method for identifying antimicrobial peptides (AMPs),^[^ ^15^ ^]^ which hold potential as anti‐infective agents. This success suggests that similar functional macro‐molecules, such as anticancer peptides (ACPs), can be discovered from metagenomic datasets. ACPs, recognized for their ability to inhibit cancer, have long been acknowledged as potent therapeutics.^[^ ^16^, ^17^ ^]^ In fact, as early as 1985, the United States Food and Drug Administration (US FDA) approved leuprolide, an ACP for use in multiple cancer indications.^[^ ^18^ ^]^ Despite decades of research and their clinical relevance, the number of clinically usable ACPs remains limited, with eight approved by the US FDA and three approved by the European Medicines Agency (EMA).^[^ ^18^ ^]^ The potential of this molecule category for the development of new cancer treatment approaches underscores the urgency of discovering novel and potent ACPs in oncology research. One strategy to achieve this goal involves chemically enhancing existing ACPs by incorporating unnatural amino acids or synthesizing the D or L isomer of an ACP to improve its drug‐like characteristics.^[^ ^19^, ^20^ ^]^ Unfortunately, the empirical discovery of novel ACPs is a highly time‐ and resource‐intensive process, necessitating the development of more efficient approaches. In recent years, several studies have explored the utility of machine learning and deep neural networks for predicting ACPs.^[^ ^21^, ^22^, ^23^, ^24^, ^25^, ^26^ ^]^ However, thus far, these studies have been limited to in silico analyses and have not been sufficiently developed to identify new ACPs from the vast and continuously expanding proteomic and genomic datasets associated with the gut microbiome.

**Figure 1:**
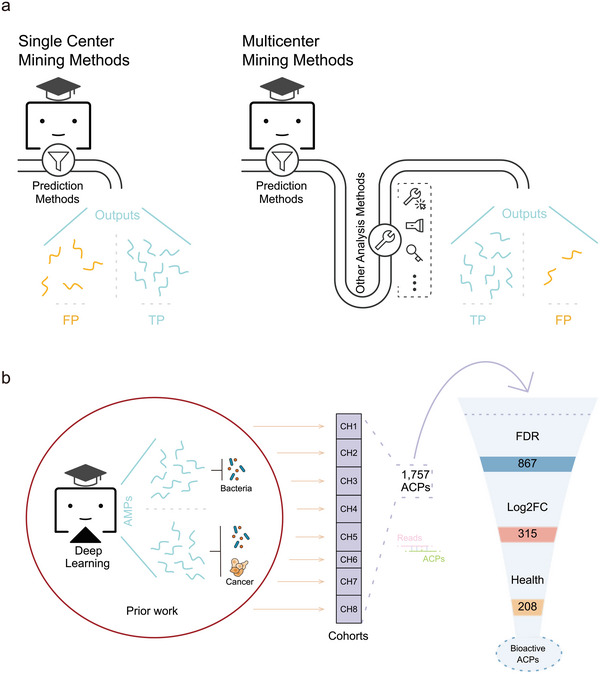
Schematic representation of the research workflow. a) A multi‐center prediction process, as opposed to a single center prediction process reliant solely on a prediction model, involves additional steps of data analysis to effectively eliminate false positives obtained from the prediction model. This process directly increases the proportion of true positives in the final prediction. b) In our study, we combined predicted potential ACPs with metagenomic cohorts analysis to screen for novel ACPs.

In this study, we employed a validated high‐throughput mining process to discover new ACPs within metagenomic datasets. Subsequently, we associated the identified ACPs with cancer phenotypes using differential analysis of gut microbiome data from multiple cohorts of CRC patients and matched healthy individuals (**Figure** **1**). Experimental validation confirmed a total of 40 candidate peptides, with 39 demonstrating functional ACP properties. Notably, two of these peptides, designated as potential ACPs (pACPs), exhibited exceptional potency, exerting robust anticancer effects across multiple cancer cell lines and significantly reducing tumor size in a subcutaneous CRC model. Despite marked differences in primary and secondary structure compared to known published ACPs, our data strongly indicate that these pACPs possess potent cancer cell‐killing capabilities. Our work expands the current understanding of ACPs and underscores the effectiveness of our approach, which combines metagenomic data analysis and mining, in identifying novel and biologically relevant peptides for further characterization and development.

## 2 Results

### 2.1 ACPs Largely Overlap with AMPs

**Figure 2:**
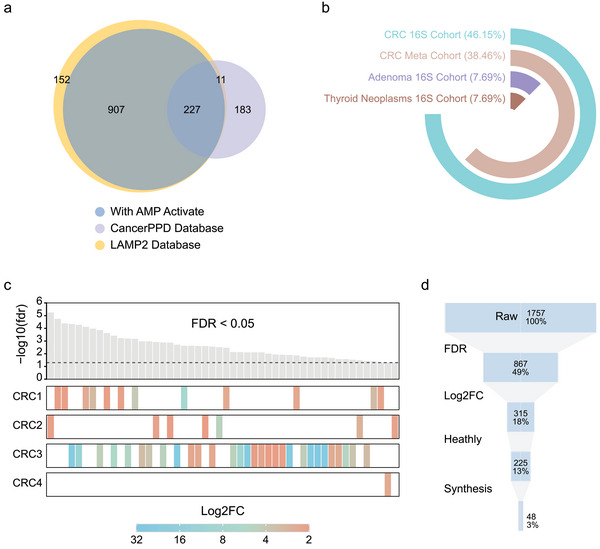
ACP screening process in our study. a) Collection of ACPs and AMPs from public databases revealed a significant overlap between them. b) Composition of metagenomic cohorts of cancer patients from public databases, with subsequent analysis focusing on cohorts containing CRC and healthy controls. c) Enrichment of pACPs in healthy controls and information regarding their source cohorts. The top panel indicates the significance of enrichment (−log10(*fdr*)) for the 40 peptides synthesized in this study (see Experimental Section), while the bottom panels display the fold changes of enrichment in healthy individuals compared to CRC patients. Each synthesized ACP is represented by a bar, with different colors indicating varying levels of enrichment in healthy individuals. CRC1: PRJDB27928; CRC2: PRJDB6070; CRC3: PRJNA397219; CRC4: PRJDB7774. d) Overview of the peptide filtering process and the number of peptides retained at each step of data analysis.

Previous studies have classified ACPs into two main types based on their targets: those inhibiting both cancer cells and bacteria, and those targeting cancer cells, bacteria, and normal cells.^[^ ^27^ ^]^ To investigate the relationship between known ACPs and AMPs, we examined the association using the CancerPPD^[^ ^28^ ^]^ and LAMP2^[^ ^29^ ^]^ databases. CancerPPD specifically collects ACPs and currently contains 3491 ACPs, of which 421 ACPs are composed entirely of natural amino acids (AAs). LAMP2, on the other hand, focuses on AMPs and currently annotates 1327 peptides as having anticancer activity, with the majority (1297 peptides) composed of natural AAs. After removing duplicates between the two databases, we identified 1480 unique ACPs. Remarkably, 1134 of these unique ACPs (76.6%) also exhibit antimicrobial activities (**Figure** **2**a), indicating a substantial overlap between ACPs and AMPs. Leveraging this overlap, we tested our deep learning‐based AMP prediction pipeline and found that it efficiently recalled the published ACPs. To avoid bias, we excluded the peptides used for model construction, resulting in 1279 remaining ACPs. Employing prediction models designed for AMPs, we successfully identified 1033 ACPs from the pool of 1279 ACPs, achieving a recall rate of 80.77%. Notably, our previous work on predicting potential AMPs^[^ ^15^ ^]^ encompassed 2349 peptides, which is likely to contain a significant subset of ACPs. These peptides can be further filtered using metagenomic data analysis.

### 2.2 Mining pACPs Using Meta‐Analysis of Metagenomic Cohorts

To filter potential peptides, we employed metagenomic data analysis, assuming that there exist significant associations between peptide abundance and specific phenotypes. For instance, in AMP detection, the network assumes that functional AMPs would exhibit a negative association with bacterial abundance. Similarly, we hypothesized that ACPs would be associated with the absence of cancer. By introducing a second “enter of mining,” this approach can potentially enhance the overall success rate of discovering functional peptides from a vast reservoir. To implement this, we collected cancer‐related metagenomic studies from GMrepo^[^ ^30^ ^]^ and selected six metagenomic cohorts comprising patients with CRC (Figure 2b). Additionally, we manually obtained two independent cohorts, resulting in a total of 496 CRC patients and 509 healthy individuals (Table S3, Supporting Information).^[^ ^31^, ^32^, ^33^, ^34^, ^35^, ^36^, ^37^, ^38^ ^]^

Next, we aimed to establish a collection of peptides showing differential distribution between CRC patients and healthy subjects across multiple cohorts. We examined the associations between peptides in the gut metagenome and the presence or absence of CRC within the CRC dataset. We calculated the relative abundance of 2349 peptides in each of the eight cohorts within the CRC dataset. Our analysis revealed that 1757 sequences were present in CRC samples. Subsequently, we profiled the differences in peptide abundance between patients and healthy controls, identifying 867 peptides that were significantly differentially expressed in CRC patients compared to healthy controls (FDR < 0.05). These peptides were then filtered based on fold‐change between the groups (Figure 2c). Applying a log2 fold change >2 cutoff, we prioritized 315 peptides that exhibited the most significant differences between the groups. Among these, 225 peptides were significantly enriched in the healthy control samples. We further selected 48 out of the 225 peptides with a relative abundance >2*10^−4^ for subsequent experimental validation (Figure 2d).

**Figure 3:**
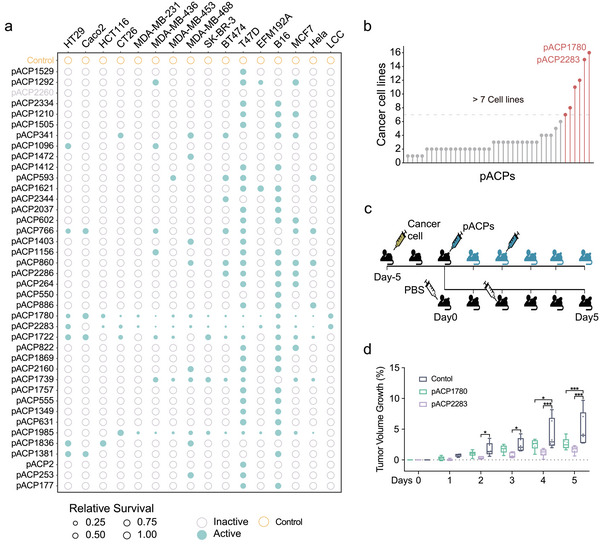
Validation of potential anticancer peptides in vitro and in vivo. a) Initial screening of anticancer activity. Individual cell lines were treated with ACPs or a vehicle (PBS), and survival rates were calculated based on the ratio of viabilities between each ACP and the vehicle (PBS). Green circles indicate positive anticancer activity, with the circle size representing the relative survival of the cancer cell lines, each test has at least three technical repetitions. b) The number of significantly inhibited cancer cell lines for each pACP. c) Experimental scheme of pACPs in a mouse model. d) Inhibitory effect of two selected anticancer peptides on tumor growth, indicated by box graphs displaying tumor sizes during the experiment. Mice treated with pACP1780 are shown in green, while those treated with pACP2283 are shown in purple. *Adjusted *P* < 0.05 and ***Adjusted *P* < 0.01 in Dunnett's test, each group contain six mice.

We successfully chemically synthesized 40 pACPs for the purpose of in vitro or in vivo characterization of their potential anticancer efficacy. In the initial step, we conducted an in vitro primary screen of these 40 pACPs using a panel of 16 different cancer cell lines, including four human CRC lines (HT29, Caco2, HCT116, and CT26), as well as 12 other cancer models (Table S4, Supporting Information). We deliberately selected a diverse panel of cell lines to assess the effect of pACPs across various genetic backgrounds. Briefly, the cancer cells were cultured in specific media (details provided in the Experimental Section) and then treated with pACPs at a final concentration of 25 µM. Peptides with minimum effective inhibition concentrations >25 µM were considered non‐ACPs. After exposure to pACPs, the survival rate of the cancer cells was measured using the MTT assay. Remarkably, 39 out of the 40 pACPs (97.5%) induced at least 20% inhibition in at least one cell line (**Figure** **3**a and Table S5, Supporting Information). Among the validated pACPs, 31 effectively inhibited proliferation in one to four cell lines, while five pACPs demonstrated significant proliferation inhibition in more than half of the tested cell lines. Thus, our method successfully identified pACPs with a very high rate of anticancer effects across various cancer cell lines.

Of the validated pACPs, pACP2283, and pACP1780 exhibited the highest potency and effectiveness against nearly all tested cell lines (Figure 3b). To evaluate the translational relevance of our methodology, we selected these two pACPs for in vivo studies. Initially, we determined the CC_50_ of pACP2283 to be 10.56 µM in CT26 cells, while for pACP1780, it was 6.23 µM (Table S6, Supporting Information). Subcutaneous tumors were established using CT26 cells in athymic nude mice, and the animals were randomized to receive two intratumoral injections of 0.80 mg kg^−1^ pACP2283, 0.51 mg kg^−1^ pACP1780, or PBS control on day 0 and day 2. The dosage of pACPs was calculated as five times the CC_50_ of each compound in CT26 cells. Tumor size was monitored daily for six days (Figure 3c). Treatment with either pACP significantly reduced tumor size compared to control‐treated animals (Figure 3d, *p* values <0.05, Table S7, Supporting Information), and no overt signs of acute toxicity were observed. Importantly, all mice survived the treatment with these two pACPs, indicating no difference in mortality between the treatment groups.

Furthermore, we investigated the safety of pACP2283 and pACP1780 both in vivo and in vitro. Initially, we treated the HEK293 cell line, representing non‐cancer cells derived from embryonic stem cells, with these two pACPs and found no toxicity to HEK293 cells (Extended Data Figure 1c,d). Additionally, we conducted an acute toxicity experiment in tumor‐free mice by administering pACPs with increasing concentrations into the abdomen. After injecting pACPs up to 50 mg kg^−1^ of mice weight, no significant symptoms of illness or body weight loss were observed in the following days. Although there was a slight decrease in the growth rate of body weight in mice after injection of pACPs at 50 mg kg^−1^ (493.5 times the CC_50_ for pACP1780 and 311.0 times the CC_50_ for pACP2283), the body weight growth rate gradually recovered over the next five days (Extended Data Figures 1a and 2b). This temporary decrease in body weight growth rate may be attributed to the high concentrations of pACPs, which temporarily disturbed the mice's homeostasis. In summary, both pACP2283 and pACP1780 exhibited neither toxicity to HEK293 cells nor signs of acute toxicity in tumor‐free mice.

### 2.3 Validated pACPs Contain Novel Sequence Features

**Figure 4:**
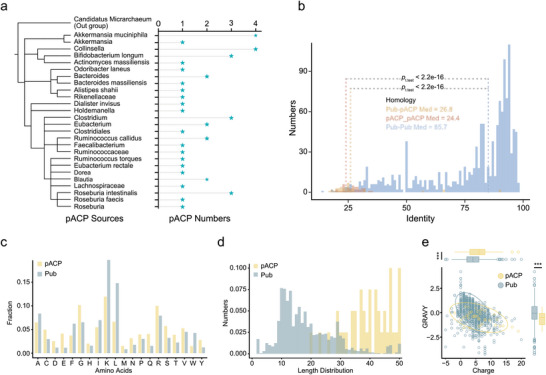
Sequence analysis of new pACPs. a) Analysis of the source bacteria of pACPs and the number of pACPs found in each bacterium. b) Distribution of sequence identities among pACPs and the published PCA set, showing significantly higher identities among published PCAs. One‐sided *t*‐tests were performed for each comparison. c) Amino acid composition of pACPs discovered in our study and known ACPs in our collected sets (pACPs in orange and published ACPs in green). d) Length distribution of pACPs and published ACPs. e) Plotting of published ACPs and pACPs based on their GRAVY Index and charge properties. Published ACPs are shown in green, and pACPs are shown in orange. Ellipses represent a 95% confidence interval assuming a distribution. The symbol “***” means *p* value < 0.01 in two‐sided *t*‐test.

Next, we conducted an investigation into the potential phylogenetic origins of the novel pACPs. Among the 39 pACPs with confirmed anticancer activity in vitro (Table S8, Supporting Information), we allocated 20 pACPs to the genomes of 12 bacterial species. Notably, *Akkermansia muciniphila*, *Bifidobacterium longum*, and *Roseburia intestinalis* were among the species with the highest number of validated pACPs (4, 3, and 3 pACPs, respectively; **Figure** **4**a). Previous reports have highlighted various beneficial health effects associated with these three species.^[^ ^39^, ^40^, ^41^, ^42^ ^]^ For instance, *Akkermansia muciniphila* has been found to be depleted in patients with hepatocellular carcinoma,^[^ ^43^ ^]^ while *Bifidobacterium longum* has been enriched in a cohort of patients with advanced non‐small cell lung cancer who responded to anti‐PD‐1 therapy.^[^ ^44^ ^]^ Additionally, a study demonstrated that oral administration of *Roseburia intestinalis* effectively inhibited solid tumor growth in mice.^[^ ^45^ ^]^ Thus, our findings, coupled with existing literature, strongly support the identification of pACPs attributed to specific microbial species, with the depletion of these ACP‐coding microbes correlating with tumor occurrence.

To gain insights into the sequence‐level features, we compared the sequences of our newly discovered pACPs with those of published ACPs in the aforementioned database. The published ACPs exhibited higher sequence identity to each other, with >80% identity observed in 64.66% of the cases. In contrast, the identity between published ACPs and our novel pACPs was predominantly <30%, and the identity between our pACPs and published ACPs ranged between 20% and 40% (Figure 4b), confirming the highly novel nature of our pACPs. Importantly, the amino acid compositions and length distribution of the pACPs differed from the published ACPs. The pACPs were relatively enriched in Asp, Glu, and polar amino acids with negative charges (Figure 4c). Furthermore, the pACPs were significantly longer (31–50 amino acids) compared to the published ACPs (6–30 amino acids) (Figure 4d). Moreover, the pACPs exhibited significantly lower Gravy index scores, which indicate average hydrophobicity and hydrophilicity (*p* = 0.00038), suggesting a higher likelihood of hydrophilicity. Additionally, the pACPs displayed a higher number of electronic charges (*p* = 0. 0041; Figure 4e).

### 2.4 The Membranes of CT26 Cell is Disrupted after Candidate pACPs Treatment

**Figure 5:**
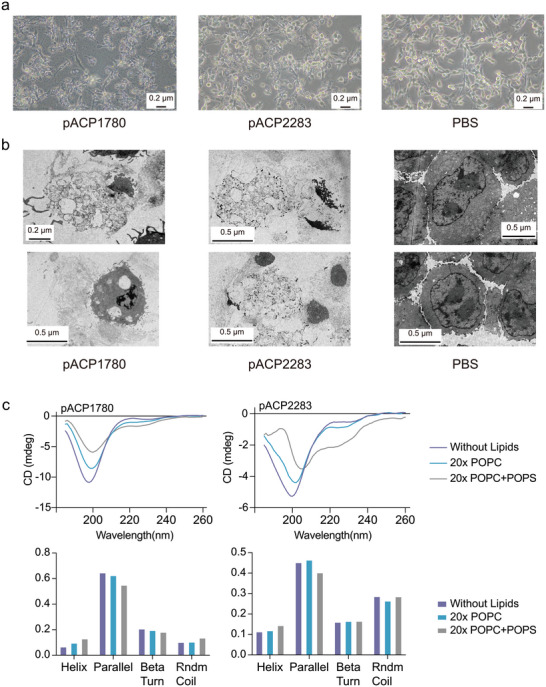
Exploring the mechanism of pACPs. a) Morphological observations of CT26 cell lines following treatment with two pACPs. b) Transmission electron microscopy (TEM) examination of CT26 cell lines treated with pACP1780 and pACP2283 at 10× CC_50_ concentration, revealing cell breakage. Experiments were performed in triplicate with similar results, and a representative figure is shown. c) CD results for the two selected ACPs and the corresponding proportions of secondary structures calculated from CD data using CDNN. Purple represents the water phase, blue represents the represents the peptide mixed with a 20‐fold POPC lipid mixture, and grey represents the peptide mixed with a 20‐fold POPC+POPS lipid mixture.

To explore the anticancer effects of pACP2283 and pACP1780 at the cellular level, we co‐cultured confluent CT26 cells with these pACPs. Using the propidium iodide (PI) assay, we observed a significantly increased fluorescence intensity,^[^ ^15^ ^]^ indicating damaged cell membranes after pACP treatments (Extended Data Figure 2). Furthermore, both pACP‐treated cultures exhibited aberrant cell morphology, and transmission electron microscopy of microscopically sliced cells revealed disintegrated cell membranes in the pACPs‐treated cells (**Figure** **5**b). Collectively, these findings suggest an association between the treatment of pACP2283 or pACP1780 and the damaged membranes of CT26 cells.

Considering that the functionality of peptides depends on their secondary structures, we investigated whether the characteristics of our pACPs contributed to their anticancer effects. Using circular dichroism (CD), we determined the potential secondary structures of the verified pACPs. The assay revealed a *β*‐parallel‐dominate structure for both pACP2283 and pACP1780. These results align with our data indicating that the newly discovered pACPs exhibit very low sequence similarity to known ACPs. Subsequently, we utilized two types of lipids to mimic the membranes of normal cells and cancer cells, and mixed them with pACP2283 and pACP1780 to evaluate the secondary structures using the CD assay once again. Specifically, we used 1‐palmitoyl‐2‐oleoyl‐*sn*‐glycero‐3‐phosphocholine (POPC) to mimic the phospholipid bilayer of normal cells, representing their more fluidic and neutral properties.^[^ ^46^, ^47^, ^48^ ^]^ In addition, we used a mixture of POPC and 1‐palmitoyl‐2‐oleoyl‐*sn*‐glycero‐3‐phosphoserine (POPS) to mimic tumor cell membranes, which possess a negative charge.^[^ ^49^ ^]^ Interestingly, the percentage of *β*‐parallel structures slightly decreased when each pACP was mixed with lipids resembling cancer cells, suggesting that the binding between pACPs and cancer cells differs from that with normal cells. Overall, we have described a novel mining methodology for discovering ACPs and demonstrated that this technique can identify potent ACPs that differ from known ACPs in terms of their primary and secondary structures.

## 3 Discussion

The identification and utilization of functional molecules from the human gut microbiome hold great promise for the development of novel therapeutics. In this study, our focus was on the discovery of novel pACPs with high efficiency from the human microbiome dataset, and we have successfully validated this approach for identifying peptides with potent anticancer effects. Previous computational methods primarily relied on prediction based on available ACPs, but there is a growing need for discovery in large and rapidly expanding biological datasets. Our study utilized existing databases to demonstrate that known ACPs largely overlap with known AMPs, allowing us to repurpose our peptide prediction process for the discovery of novel pACPs. By analyzing a gut microbiome dataset from patients with CRC and healthy control subjects, we associated the occurrence of ACPs with the presence or absence of cancer, hypothesizing that the abundance of bona fide ACPs would be negatively correlated with cancer presence. Through extensive validation, our study confirmed that over 97% of the identified pACPs (39 out of 40) exhibited anticancer effects in vitro, underscoring the high effectiveness of our multi‐center mining strategy that combines peptide prediction and metagenomic analysis of cohorts with specific phenotypes. Furthermore, the newly discovered ACPs displayed very low sequence identity with known ACPs, and the secondary structures of the two most potent pACPs were confirmed to be *β*‐parallel‐dominant.

While almost all the newly discovered pACPs demonstrated the ability to kill some cancer cells, their anticancer efficacy varied widely. In an in vitro screening of pACPs across 16 cancer cell line models, five pACPs inhibited over half of the tested cell lines. Notably, two pACPs exhibited significant inhibition in nearly all cell lines, and they were particularly potent in CT26 cultures (CC_50_ <11 µM). Importantly, although our study focused on pACPs derived from the human gut microbiome and prioritized based on a CRC dataset, several of these pACPs, including the two most potent ones, effectively inhibited breast cancer cell lines, including the challenging triple‐negative breast cancer cells. Furthermore, treatment with these two novel pACPs resulted in morphology and membrane damage in CT26 cells while sparing non‐cancerous HEK293 cells, suggesting potential selectivity of the discovered ACP candidates. We have established a link between cell membrane disruption in cancer cells and the inhibitory effects of pACPs, supported by the absence of such effects in HEK293 cells. Additionally, we observed structural changes in the peptides when exposed to lipid mixtures mimicking normal versus cancer cells, indicating that the peptides likely function through interaction with and potential disruption of membrane integrity. However, the causal relationships and temporal order between cell death and membrane breakdown are challenging to determine, necessitating further dedicated studies to establish the exact mechanisms involved.

In vivo experiments involving two intra‐tumoral injections of the two most potent pACPs demonstrated significant shrinkage of subcutaneous tumors derived from CT26 cells in athymic nude mice compared to the vehicle control, with both pACPs showing good tolerability. Enhancements in pACP delivery, such as utilizing cancer‐targeting nanoparticles, could potentially expand the therapeutic index of these agents. Further investigations are warranted to assess the long‐term tolerability, pharmacokinetics, and efficacy against other tumor types in vivo, as suggested by data from the in vitro cancer cell line screen.

In summary, we have described a multi‐center paradigm for the discovery of functional peptides from metagenomic data, effectively identifying pACPs. This approach associates peptide presence with cancer phenotypes by leveraging publicly available datasets and creating an additional “center of mining”. By utilizing an available metagenomic database, we have identified dozens of novel pACPs, significantly expanding the current collection of known ACPs, and we have demonstrated proof‐of‐concept that our technique can identify pACPs capable of suppressing tumors in vivo. This approach can be further extended by replacing the prediction method in the first center with other functionally similar methods, such as existing prediction programs or HMM methods, and by incorporating more detailed target molecule features in the second center, as demonstrated in our study based on a metagenomics cohort specifically constructed for peptide‐related analysis. Furthermore, the accumulation of proteomics and transcriptomics data is essential to fully unlock the potential of the gut microbiome in mining functional peptides, including anticancer, anti‐aging, and antimicrobial peptides. In addition to peptide mining, the scope of target molecules can be expanded to include proteins, nucleic acids (genes or functional RNAs), and more in future research endeavors.

## 4 Experimental Section

### Data Collection

The ACPs data were obtained from CancerPPD^[^ ^28^ ^]^ and LAMP2^[^ ^29^ ^]^ databases, encompassing a comprehensive collection of ACPs as of 13th September 2020. To ensure natural amino acid composition, the ACP sequences containing non‐natural amino acids were excluded from both databases, resulting in a final set of 1480 unique ACPs. To assess the relative abundance of pACPs, the data were collected from a gut microbiology cohort of CRC patients downloaded from NCBI as of March 2021. The 16S rRNA data‐only cohorts and derived eight cohorts were excluded from various independent studies in Eurasia, comprising a total of 1005 samples, including 496 CRC patients and 509 healthy controls.^[^ ^31^, ^32^, ^33^, ^34^, ^35^, ^36^, ^37^, ^38^ ^]^ The set of pACPs sequences consisted of 2349 peptides with potential antimicrobial activity and coding evidence obtained from a previous study.^[^ ^15^ ^]^

### pACPs Profiling in Gut Metagenomes

To determine the abundance of pACPs in each metagenomic sample, PALADIN software (version 1.4.0)^[^ ^50^ ^]^ was employed to align pACPs sequences with metagenomic reads. Subsequently, SAMtools (version 1.7)^[^ ^51^ ^]^ was used to calculate the abundance of pACPs. The functions of SAMtools used included “ort”,’ “index,” and “idxstats.” Abundance was calculated by determining the coverage per million reads.

### Distribution Analysis of ACPs across Cohorts

A two‐tailed Wilcoxon rank‐sum test using the “wilcox.test” function from the *R* package “stats” (version 3.4.4) was performed to assess differences in pACPs abundance. To control the false discovery rate, the “p.adjust” function was used to adjust the *p*‐values using the Benjamini and Hochberg method or FDR (false discovery rate). A threshold of transformed FDR values was defined at 0.05, and log2 fold change (Log2FC) was calculated in *R*, with a cutoff set at 2.

### Initial Screening of Anticancer Activities in Cell Lines

To evaluate the activity of the 40 potentially effective pACPs, a panel of 16 cancer cell lines was treated, including human CRC cell lines (HT29, Caco‐2, HCT116). Details of the cell lines used, respective media, and culture conditions were presented in Table S1 (Supporting Information). Once the cells reached 60% confluence, it was incubated with each candidate peptide at a concentration of 25 µM pACPs for 24 h. Concurrently, the complete medium was replaced with a maintenance medium containing only 1% FBS. Subsequently, the viability of each cell line using the MTT Cell Proliferation and Cytotoxicity Assay Kit according to the manufacturer's instructions was assessed. Cell growth was quantified, and candidate peptides demonstrating a growth inhibition rate ≥20% were identified as effective pACPs with anticancer activity. For the CRC cell line CT26, it was calculated the CC_50_ value using two peptides (pACP1780 and pACP2283) with relevant data presented in Table S6 (Supporting Information). The calculation was performed using an online tool: https://www.aatbio.com/tools/ic50‐calculator.

### Animal Experiment

Six‐week‐old female nude mice were obtained from Beijing HFK Bioscience Co., Ltd. and were acclimated to the laboratory environment for one‐week prior to the commencement of the experiments. All experimental procedures were conducted in a manner that minimized discomfort, distress, and pain to the animals. Mice were group‐housed, with a maximum of five animals per cage, under a light‐dark cycle of 12 h, with a constant temperature of 21–22°C and humidity of 55 ± 5%. They were provided with standard rodent chow and water ad libitum. The Ethics Committee of the Institute of Microbiology, Chinese Academy of Sciences approved all procedures (SQIMCAS2021005).

### Xenograft Tumor Model

Murine colorectal carcinoma cell line CT26 was cultured in RPMI‐1640 medium supplemented with 10% fetal bovine serum and 1% penicillin/streptomycin at 37°C and 5% CO2. Tumor formation was induced by injecting was 5×106 ml^−1^ CT26 cells into the right forelimb of each mouse. All mice were housed in the same environment with sufficient food and water. Throughout the study, the survival status and tumor progression of the mice on a daily basis were observed. The mice were randomly divided into three groups and received different treatments. CT26 cells were injected on Day 5 for all groups. On Day 0, ACPs (pACP1780: 0.51 mg kg^−1^ and pACP2283: 0.80 mg kg^−1^) were intratumorally injected, while the Control group received an intratumoral injection of the same volume of PBS. On Day 2, the ACPs were intratumorally injected again, and the Control group received an injection of the same volume of PBS.

### Toxicity Detection

To assess the toxicity of pACP1780 and pACP2283 at the cellular level, HEK293 cells were grown to 60% confluence in complete medium (10% FBS) for 24 h, and then treated with increasing concentrations (1.5, 3, 6, 12.5, 25, 50, and 100 µM) of each pACPs in maintenance medium (1% FBS) for 24 h. The viability of HEK293 cells was determined using the MTT Cell Proliferation and Cytotoxicity Assay Kit following the manufacturer's instructions. For acute toxicity in tumor‐free mice, the pACPs were injected into the abdomen at increasing concentrations of 2, 10, and 50 mg kg^−1^ of mouse weight over three days, and the body weights were recorded throughout the process.

### Sequence Similarity Estimation

The Needleman–Wunsch algorithm implemented in the “needleall” function from the EMBOSS software package (version 6.6.0.0) was used to estimate the similarity between the pACPs and published ACPs. The similarity was calculated by counting the number of identical amino acid pairs in the alignment. Default parameters were used, and the “identity” parameter was excluded from the graph.

### Amino Acids Frequencies

The percentage of each amino acid in the peptides was calculated using the “ProteinAnalysis” function imported from the Biopython module Bio.SeqUtils.ProtParam (version 1.75).

### Peptide Synthesis

The peptides used in this study were synthesized by Royo Biotech using solid‐phase peptide synthesis. The accurate molecular weights of the peptides were determined by mass spectrometry. The purity of all peptides was assessed by high‐performance liquid chromatography, and the purity of all peptides exceeded 90%.

### Phylogenetic Tree

The phylogenetic tree was constructed based on the NCBI taxonomy section using the NCBI ID of individual species. *Candidatus Micrarchaeum* was included as an outgroup. The taxonomy ID of potential source bacteria could be found in Table S2 (Supporting Information).

### Gravy and Charge

In this study, Gravy index scores were utilized, which represented the average hydrophobicity and hydrophilicity, instead of directly calculating the hydrophobicity of individual peptides. The Gravy index was obtained by applying the “ProteinAnalysis” function to each peptide, which was imported from the Biopython module “Bio.SeqUtils.ProtParam” (version 1.75).^[^ ^52^ ^]^ Additionally, it was determined the charge of each peptide using the AntiCP 2.0 prediction tool.^[^ ^53^ ^]^

### Transmission Electron Microscopy

First, CT26 cancer cells were treated with peptides pACP1780 and pACP2283 for two hours. The digested CT26 cancer cells were then resuspended in double‐strength fixative, which consisted of 2% (w/v) paraformaldehyde and 2.5% (v/v) glutaraldehyde in 10 mM HEPES buffer (pH 7.0). Subsequently, the cells were incubated for 2 h at room temperature on a roller and stored at 4°C. Afterward, the fixed cells were pre‐embedded with agar, subjected to four washes with 5 mM HEPES buffer (pH 7.0) (5 min per wash), and post‐fixed with 1% osmium tetroxide (OsO4) for 30 min. Following post‐fixation, the specimens were rinsed twice with water and dehydrated using an acetone series (5 min each; 30%, 50%, 70%, 85%, 90%, 3×5 min 100%). Subsequently, the specimens were infiltrated with a series mixture of acetone and Spurr's resin (3:1 for 45 min, 1:1 for 1.5 h, and 1:3 for 2 h) and finally embedded in Spurr's resin. Ultrathin sections with a thickness of ≈70 nm were stained with 2% uranyl acetate and lead citrate. Electron micrographs were captured using a JEM‐1400 transmission electron microscope (JEOL, Japan).

### Circular Dichroism (CD) Spectroscopy

CD measurements were conducted using a Chirascan spectrometer (Applied Photophysics Ltd., UK) with quartz cells having a path length of 1 mm at 25°C. Scans were performed in the wavelength range of 190–260 nm with a bandwidth of 0.5 nm, 0.5 nm step resolution, 100 nm min^−1^ scan speed, and 2s response time. To prepare the vesicle sample, 1‐palmitoyl‐2‐oleoyl‐*sn*‐glycero‐3‐phosphocholine (POPC) and 1‐palmitoyl‐2‐oleoyl‐*sn*‐glycero‐3‐phosphoserine (POPS) were dissolved in a mixture of chloroform and methanol (3:1 v/v) to obtain a POPC/POPS (4:1 mol mol^−1^) solution. This solution was evaporated under a gentle stream of nitrogen and then kept under vacuum overnight. Simultaneously, a solution containing only POPC was prepared to mimic the cell membrane of normal cells. The dried lipid film was resuspended in 10 mM phosphate buffer (pH 7.0) by vortexing, resulting in a concentration of 0.8 mM, followed by sonication for 20 min in a high‐power ultrasonic bath. Different peptide solutions in 10 mM phosphate buffer (pH 7.0) were titrated with 0.8 mM DMPE/DMPG to achieve a peptide‐lipid ratio of ≈1:20. The CD spectra were recorded for the peptide (final concentration of 40 µM) in phosphate buffer alone or in the presence of DMPE/DMPG. The spectra were processed using a Savitsky–Golay smoothing filter, and the content of secondary structures was estimated using CDNN software (version 2.1).

### Cell Viability Assay

Cell viability was assessed using propidium iodide (PI, ZS822‐1) according to the manufacturer's instructions. Briefly, CT26 cells were seeded in a 96‐well plate at a density of 1×104 cells per well. After 24 h, pACP1780 and pACP2283 were added to the wells at concentrations of 6.23 and 10.56 µM, respectively. The plate was then incubated for 1, 2, 6, and 12 h. Subsequently, the cells were cultured in medium containing 10 µg ml^−1^ PI for 45 min. The fluorescence intensity was measured using a CytationTM 5 instrument (Bio Tek) with excitation/emission wavelengths of 535/617 nm.

### Statistics Analysis

Statistical data were analyzed via *R* (version 4.2.2) and GraphPad Prism 9.4.1. All bar plots were presented as mean ± SEM. For comparisons within in vivo and in vitro experiments data, Dunnett's test were used, and an adjusted *P* values < 0.05 was considered statistically significant. Benjamini & Hochberg (alias FDR) method were used for *P* value adjusted. The statistical analyses and sample size applied for each experiment were indicated in the figure legends.

## Conflict of Interest

The authors declare no conflict of interest.

## Supporting information

Supporting Information

## Data Availability

The data that support the findings of this study are available from the corresponding author upon reasonable request.
